# Not only being compliant, but also being constant in brace wearing improves short-term results: a prospective Thermobrace study

**DOI:** 10.1186/1748-7161-8-S2-O55

**Published:** 2013-09-18

**Authors:** Sabrina Donzelli, Monia Lusini, Salvatore Minnella, Fabio Zaina, Stefano Negrini

**Affiliations:** 1 ISICO Italian Scientific Spine Institute, Milan, Italy

## Background

Compliance, together with quality of bracing, have shown to achieve good results in scoliotic patients. However, nothing is known about constant brace wearing (CBW), which means maintaining the same number of hours almost every day. CBW is recommended at each brace prescription, but is it true that being constant will bring the best results? 

## Purpose

The goal of this study was to find out if CBW is rewarded with better bracing results in the short term. 

## Methods

Prospective controlled cohort study nested into a clinical database started in 2003. On December 31, 2012, out of 11,800 patients in the database, we selected 168 who met the following inclusion criteria: Adolescent Idiopathic Scoliosis, Sforzesco brace prescription 18 to 23 hours/day, at least four months of observation, Thermobrace (TB) adoption and out-of-brace x-ray before treatment. CBW (104 patients) was compared to fickle-brace wearing (FBW: 64): due to the abnormal distribution of TB values, one hour in the inter-quartile range distinguished the two groups. A 6-degree Cobb cut-off was defined to classify results as improved, worsened or stabilized. Patients were also classified for compliance: High (HC: >95% of prescription), Middle (MC: 70-94%) and Low (LC: <70%). For statistical analysis, chi-square test has been used; the relative risk (RR) of not improvement (i.e., progression or stabilization only) with 95% Confidence Interval (IC95) has been calculated as well.

## Results

Males were more frequent in FBW (78.1% vs 12.5% in CBW – P<0.0001). CBW-HC showed a high percentage of improved and stabilized curves (44.2% and 28.8% ) when compared to FBW (10.9% and 10.9%). CBW are frequently HC, 81.7% versus 21.8% in FBW (P<0.0001). In both groups, HC were more frequent in the group of prescription over 22h per day, while the severity of scoliosis did not affect compliance or CBW. Without distinctions for compliance, RR in the short-term for FBW was 1.35 (IC95: 0.95-1.93 – P=0.08). FBW-MC/LC, compared to CBW-HC showed a RR of 1.50 (IC95: 1.0-2.3 - P=0.04). 

## Conclusions and discussion

Wearing a brace rigorously (HC) and constantly (CBW) provides good results, and this remains true in MC. Compliance to prescription is fundamental to a higher probability of achieving good results, but FBW can represent a risk for progression. Future studies should document these data at the end of treatment. 
